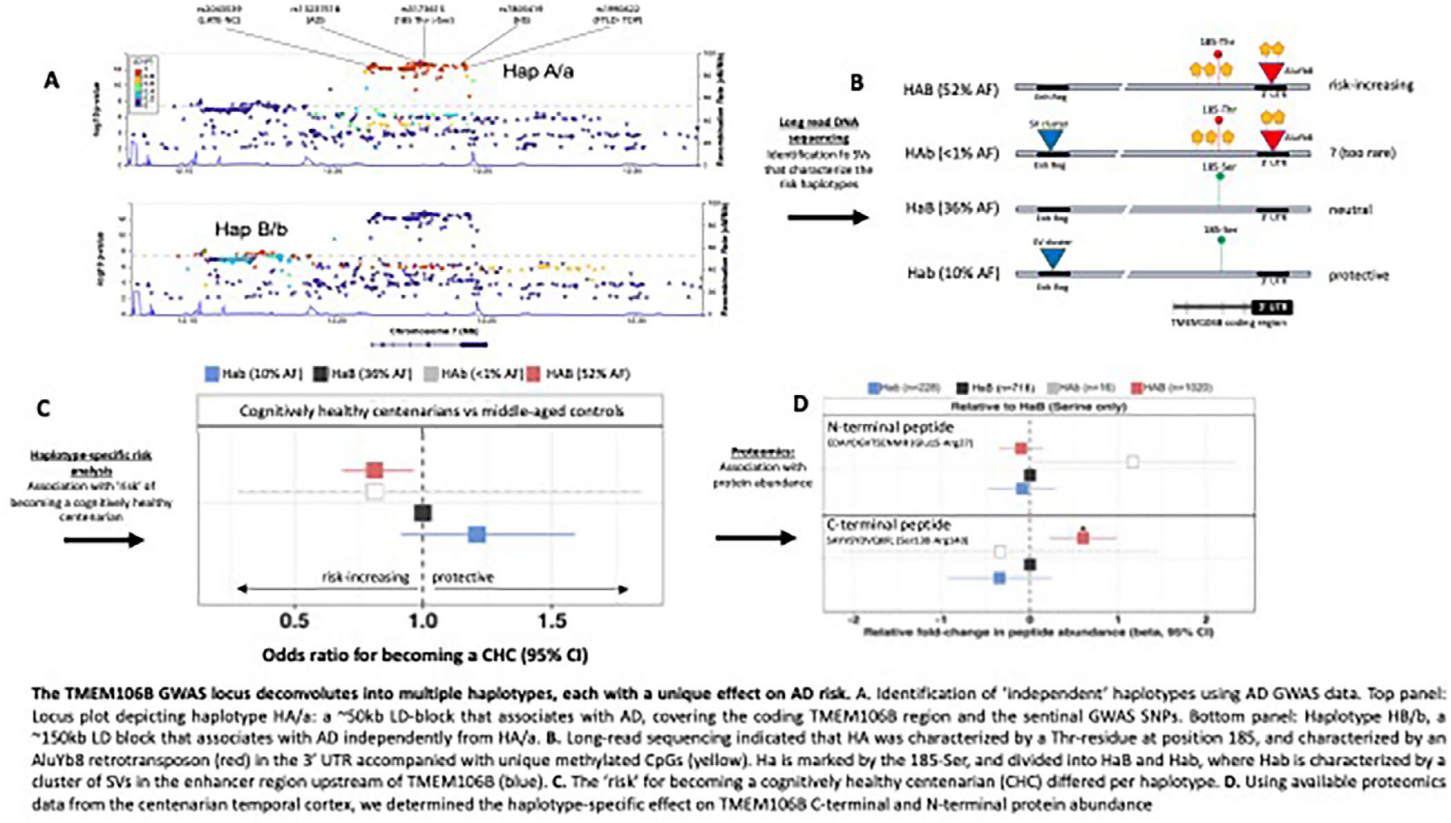# Fine‐mapping of the TMEM106B locus reveals four haplotypes that are differentially associated with risk for neurodegeneration

**DOI:** 10.1002/alz70855_104407

**Published:** 2025-12-24

**Authors:** Henne Holstege, Alex N Salazar, Lydian Knoop, Yolande A.L. Pijnenburg, Sven J van der Lee, Sanduni Wijesekera, Jana Krizova, Mikko Hiltunen, Markus Damme, Leonard Petrucelli, Marcel JT Reinders, August B. Smit, Marc Hulsman

**Affiliations:** ^1^ VIB‐KU Leuven Center for Brain & Disease Research, Leuven, Belgium; ^2^ Genomics of Neurodegenerative Diseases and Aging, Human Genetics, Amsterdam UMC, Amsterdam, Netherlands; ^3^ Alzheimer Center Amsterdam, Neurology, Amsterdam UMC, Amsterdam, Netherlands; ^4^ University of Eastern Finland, Kuopio, Finland; ^5^ Christian‐Albrechts‐Universitaet Kiel, Kiel, Germany; ^6^ Mayo Clinic, Jacksonville, FL, USA; ^7^ Delft University of Technology, Delft, Netherlands; ^8^ Center for Neurogenomics and Cognitive Research, Vrije Universiteit Amsterdam, 1081HV, Amsterdam, Netherlands

## Abstract

**Background:**

Genome‐wide association studies (GWAS) linked *TMEM106B* variants to susceptibility for neurodegenerative diseases, but the causal genetic elements remain unclear.

**Method:**

We used genotyping data from 5,792 Alzheimer disease cases and controls, and applied COJO to identify haplotypes in the TMEM106B locus that independently associated with AD. Then, we used long‐read sequencing data from 513 individuals to annotate these haplotypes with structural variations that map into them.

**Results:**

Analysis of the genotyping data revealed that the *TMEM106B* locus consists of four major haplotypes: HA/Ha (covering the coding region), and HB/Hb (covering the upstream regulatory region). These combine into four combinations with varying population‐frequencies: *HAB* (57%), *HaB* (34%), *Hab* (9%), and *HAb* (<1%). Long‐read sequencing of 513 individuals showed that *HA* haplotypes (marked by 185‐Threonine) carry unique methylated CpG sites and an AluYb8‐retrotransposon in the 3' UTR, while the *Ha* haplotypes are marked by the 185‐Serine allele. *Hb* haplotypes carry several structural variants (SVs) in nearby distal enhancers, including a 19 Kbp rearrangement, absent in all other haplotypes. Joint association models revealed that the *HAB* combination (AluYb8+185‐Threonine) is risk‐increasing, while *Hab* (SVs+185‐Serine) confers the protective effect. *HaB* (185‐Serine only) is neutral, while *HAb* was too rare to assess. Relative to middle‐aged non‐demented controls, cognitively healthy centenarians were more enriched with *Hab* (OR=1.49, padj=2.18×10‐2) than with *HaB* (OR=1.23, padj=5.06×10‐2). Proteomic analysis of temporal cortex tissues (*n* = 182) indicated that relative to the neutral *HaB* combination, the protective *Hab* is associated with 1.1‐fold *lower* TMEM106B C‐terminal peptide abundance, while the risk‐increasing *HAB* is associated with 1.16‐fold *higher* abundance.

**Conclusion:**

Our data indicates that the genetic structure underlying the association of the *TMEM106B* locus with neurodegenerative diseases is driven by the effect of multiple haplotypes.